# New Perspectives on Antimicrobial Agents: Fosmanogepix

**DOI:** 10.1128/aac.00324-26

**Published:** 2026-06-04

**Authors:** Frederic Lamoth

**Affiliations:** 1 Infectious Diseases Service, Department of Medicine, Lausanne University Hospital and University of Lausanne 30639, Lausanne, Switzerland; 2 Institute of Microbiology, Department of Laboratory Medicine and Pathology, Lausanne University Hospital and University of Lausanne 536517, Lausanne, Switzerland; Samsung Medical Center Department of Infectious Diseases, Seoul, Republic of Korea

**Keywords:** APX001, Gwt1, *Fusarium*, *Scedosporium*, *Lomentospora*

## Abstract

Invasive fungal infections (IFIs) are associated with high mortality rates and pose important therapeutic challenges because of the limited number of antifungal drug classes, their toxicity, and the emergence of resistance. Novel first-in-class antifungal agents have been recently approved (ibrexafungerp) or are currently being tested in clinical trials and are available via expanded access programs (olorofim, fosmanogepix). Among them, fosmanogepix (a prodrug of manogepix) represents a promising future therapeutic option for many IFIs because of its broad-spectrum antifungal activity and its favorable pharmacologic properties, such as oral bioavailability, extended tissue distribution (including the central nervous system), a good safety profile, and the absence of relevant drug-drug interactions. Fosmanogepix may become an interesting alternative option for the treatment of invasive candidiasis due to multidrug-resistant isolates (e.g., *Candidozyma auris*) or invasive aspergillosis due to azole-resistant *Aspergillus* spp. It also demonstrated very encouraging results for the treatment of invasive fusariosis and other refractory mold infections (e.g., scedosporiosis, lomentosporiosis). Novel gepix compounds have been developed and could make their niche in the future (e.g., APX2039, prodrug: APX2096, for cryptococcal meningitis, and APX2020, prodrug: APX2097, for coccidioidomycosis). This review discusses the future place of fosmanogepix in the antifungal armamentarium against IFIs.

## PERSPECTIVE

Invasive fungal infections (IFIs) are life-threatening diseases affecting individuals with critical conditions or severe immune defects. Their prevalence is increasing along with the development of novel anti-cancer therapies and the continuous expansion of patients with chronic immunosuppressive conditions and prolonged life expectancy (1, 2). IFIs are diagnosed in about 5%–10% hematologic cancer patients, with overall mortality rates of about 40%–50% (3–5). Similar rates are observed in ICUs (6, 7). Invasive candidiasis (IC, caused by *Candida* spp.) and invasive aspergillosis (IA, caused by *Aspergillus* spp.) represent the majority of IFIs in the United States and Europe. Cryptococcosis (*Cryptococcus* spp.) and *Pneumocystis* pneumonia are responsible for high morbidity and mortality in regions with high prevalence of human immunodeficiency virus infection (1). Until recently, the antifungal armamentarium was limited to mainly three drug classes: the polyenes (essentially amphotericin B formulations), the triazoles, and the echinocandins. The burden of IFI and the increasing use of antifungal drugs have resulted in higher rates of antifungal resistance, especially among *Candida* species (8). Moreover, clones of azole-resistant *Aspergillus fumigatus* have developed with the use of fungicides in agriculture (9). The use of mold-active azole prophylaxis in high-risk hematologic cancer patients has also been associated with a shift from *Aspergillus* toward more resistant mold species causing IFI (10, 11). In addition to the selective pressure of antifungals, other factors, such as global warming, may contribute to the emergence of novel pathogenic species. Such an example is well illustrated by *Candidozyma* (formerly *Candida*) *auris*, a yeast capable of developing multiple resistances, which was first described in 2009 and has spread on all continents (12, 13). The awareness of the medical community about the threat of fungi and the need for novel antifungal therapies has increased during the last decade. In 2022, the World Health Organization (WHO) published a priority list of fungal pathogens deserving particular attention for research and development (14). Recently, several novel antifungal agents (e.g., ibrexafungerp, olorofim, fosmanogepix, rezafungin, tetrazoles, lipid nanocrystal amphotericin B) have been developed and tested in clinical trials with promising results (15, 16). The present review will focus on fosmanogepix (FMGX), a first-in-class antifungal agent with interesting pharmacologic properties and broad-spectrum antifungal activity.

## MECHANISM OF ACTION

Manogepix (MGX, formerly referred to as E1210 or APX001A), the active moiety of FMGX (APX001), targets Gwt1, an enzyme localized in the endoplasmic reticulum and involved in the biosynthesis pathway of glycosylphosphatidylinositol (GPI)-anchored mannoproteins (transfer of an acyl group) (17, 18). MGX competitively inhibits the binding of the Gwt1 substrate palmitoyl-CoA (19). GPI-anchored proteins are essential for cell wall integrity and play important roles in adhesion and escape from the host immune system (20, 21). For increased solubility and bioavailability, MGX is administered as a prodrug, FMGX, which is rapidly transformed by systemic alkaline phosphatases into the active compound (17). It is fungal-specific with no effect on the human Gwt1 ortholog (PIG-W) but demonstrated some *in vitro* activity against the protozoan parasite *Trypanosoma cruzi* (18, 22).

Studies with *Candida albicans* showed that MGX had excellent activity against planktonic and sessile cells, with inhibitory effects on germ tube formation, adherence to inert surfaces, and biofilm formation (18). It displayed potent fungistatic activity with absence of trailing growth but was not fungicidal (23). A prolonged post-antifungal effect (PAE) was observed both *in vitro* and *in vivo* (17). Against *A. fumigatus*, manogepix causes little cell lysis and induces aberrant growth morphology comparable to that observed with echinocandins (23, 24). A compensatory effect on the cell wall with increased chitin content has been observed in both *C. albicans* and *A. fumigatus* (24, 25).

## *IN VITRO* ACTIVITY

Antifungal susceptibility testing of MGX against relevant pathogenic fungal species showed good *in vitro* activity against most of them. Results are usually interpreted as the minimal inhibitory concentration (MIC) causing ≥50% inhibition for yeasts and minimal effective concentration (MEC) causing significant morphological change (blunting) for molds (26). For the most relevant *Candida* spp., MIC encompassing 90% of isolates (MIC_90_) were 0.008–0.12 µg/mL, except for *C. krusei*, *C. kefyr,* and *C. inconspicua* (MIC >0.5 µg/mL) (26–31). Notably, constantly low MICs (≤0.06 µg/mL) were observed among *C. auris* isolates (32–34). *Cryptococcus neoformans* displayed large inter-isolate variability (MIC range 0.015 µg/mL–4 µg/mL, MIC_90_ 0.5 µg/mL–1 µg/mL) (26, 29–31, 35). For *Aspergillus* spp., MEC_90_ ranged from 0.008 to 0.06 µg/mL (including azole-resistant *A. fumigatus* isolates and most cryptic species) (26, 29–31, 36–38). MGX showed consistently good activity against *Fusarium solani* complex (MEC ≤0.12 µg/mL) and variable activity against other *Fusarium* spp. (notably *F. oxysporum* and *F. verticilloides*) (MEC ranges ≤0.015 µg/mL–16 µg/mL) (26, 29–31, 36, 38–40). Activity against *Scedosporium* spp. and *Lomentospora prolificans* was good (MEC_90_ ≤0.12 µg/mL), although some isolates (*Scedosporium apiospermum* and *L. prolificans*) with high MEC (16 µg/mL) have been reported (26, 29–31, 38, 40). Activity against *Mucorales* is modest (MEC_90_ 4 µg/mL–8 µg/mL for most species, except for some isolates exhibiting lower MEC (0.25 µg/mL–1 µg/mL) (26, 29–31, 38). Testing of rare molds and yeasts (limited data set) suggests good activity against *Magnusiomyces* spp., *Rhodotorula* spp., *Saccharomyces cerevisiae*, *Aureobasidium* spp., *Exophilia* spp., *Hormographiella aspergillata*, *Scopulariopsis* spp., *Purpureocillium lilacinum*, *Paecilomyces variotii*, *Rasamsonia argillacea*, *Penicillium* spp., *Trichoderma* spp., *Sarcocladium* spp., *Phaeoacremonium* spp. (MIC/MEC_90_ ≤0.06 µg/mL) and modest activity against *Trichosporon* spp. and some black molds (*Curvularia* spp., *Alternaria alternata*, *Verruconis galopava*, MEC ≥0.25 µg/mL) (26, 29–31, 38). Excellent *in vitro* activity against dimorphic fungi (*Coccidioides immitis*/*posadasii*, *Blastomyces* spp., *Emergomyces* spp., *Sporothrix* spp.) was also observed (41–43).

*In vitro*, MGX showed synergism with anidulafungin against some *C. auris* isolates and with calcineurin inhibitors against *C. albicans*, *A. fumigatus,* and *C. neoformans* (25, 44). Positive interactions were also observed with fluconazole against *C. neoformans* and itraconazole against *Madurella mycetomatis* (35, 45).

## EFFICACY IN ANIMAL MODELS

The efficacy of FMGX has been tested in different murine models of IFIs. Both the intravenous and oral routes of administration have been used in these models. A study in rats and monkeys showed excellent absorption by both routes, distribution in all organs and tissues, including the brain, eye, and urine, and primary elimination by biliary/fecal excretion (46). It is noteworthy that the MGX half-life is shorter in mice than in humans and that the use of a cytochrome P450 inhibitor was shown to improve exposure and mimic human conditions, although this strategy was not systematically applied (47).

In mice, FMGX was as effective as conventional antifungal therapies in treating IC and was also very effective against *C. albicans* azole- or echinocandin-resistant and *C. auris* infections (47–51). The pharmacokinetic/pharmacodynamic (PK/PD) index best correlating with efficacy was the 24 h free-drug concentration-time curve (AUC)/MIC ratio (*f*AUC_0-24_/MIC), with some species-specific PK/PD targets (i.e., lower for *Candida glabrata* compared to *C. albicans* and *C. auris*) (52). FMGX also achieved significant reduction of *Candida* burden in targeted organs, including the brain (48, 51). It was superior to micafungin for penetration and reduction of fungal burden in liver abscess (53). In a rabbit model of *Candida* endophthalmitis, FMGX achieved effective concentrations in the eye and central nervous system (CNS) (54). FMGX was also tested in a murine model of oropharyngeal candidiasis and showed similar efficacy to fluconazole (49). In a murine model of cryptococcal meningitis, FMGX alone could significantly decrease the fungal burden in lung tissue, and its combination with fluconazole was more effective than either monotherapy in the brain (35).

In neutropenic murine IA, FMGX demonstrated a significant impact on survival and reduction in lung fungal burden with the AUC/MEC index best correlating with efficacy (55–57). Its efficacy was comparable to that of liposomal amphotericin B or posaconazole (55, 58). FMGX was as effective as high-dose liposomal amphotericin B in treating disseminated fusariosis (*F. solani*) and superior to comparative drugs for invasive scedosporiosis (*S. apiospermum*) in neutropenic mice (58, 59). Despite modest *in vitro* activity against Mucorales, FMGX demonstrated significant and comparable efficacy to isavuconazole and liposomal amphotericin B in a neutropenic murine model of *Rhizopus arrhizus* infections irrespective of the MEC (58, 60). Notably, FMGX achieved a reduction in brain fungal burden that was similar to isavuconazole (60). Interestingly, despite the absence of *in vitro* synergism, the combination of FMGX and liposomal amphotericin B was significantly superior to either monotherapy in murine models of IA (*A. fumigatus*), mucormycosis (*R. arrhizus*), and fusariosis (*F. solani*) (58).

Finally, FMGX was tested successfully in a murine model of pulmonary coccidioidomycosis (43).

## PHASE I STUDIES

FMGX PK in humans was linear (dose-proportional), with excellent oral bioavailability (90%–100%) and maintenance of appropriate dose exposure while switching from intravenous to oral formulation (61, 62). Exposure was not affected by food but was better tolerated when administered after a meal (62). Mean half-life (t½) was about 2 days (61, 63). Elimination was equally distributed between urine and biliary/fecal routes, with diverse metabolic pathways (64). Safety studies in healthy volunteers and patients with acute leukemia and neutropenia showed good tolerability with mild adverse events, which were more frequent at high dosages and consisted mainly of gastrointestinal disorders (nausea or vomiting), followed by neurologic/psychiatric disorders (headache, dizziness, delirium) (61–63). Steady state of the drug was reached between days 2 and 4 (61). Based on these data, the proposed regimen to achieve targeted concentrations and good tolerability was 1,000 mg bid as intravenous loading dose (day 1), followed by 600 mg qd or 800 mg qd for the intravenous and oral dosing, respectively (61). Drug-drug interaction study with a CYP3A4 inhibitor (itraconazole) and a pan-cytochrome P450 (CYP) inducer (rifampicin) showed no or moderate effect (45% decrease) on MGX exposure, respectively (65). Whether FMGX dosing adjustment or MGX therapeutic drug monitoring may be required in case of co-administration with a CYP inducer should be further investigated. Inversely, there is currently no evidence that FMGX affects the bioavailability of drugs metabolized by CYP.

## PHASE II / III AND OTHER CLINICAL STUDIES

To date, three open-label single-arm phase II trials have been completed and published (Table 1). In one study, 20 non-neutropenic patients with candidemia (any *Candida* spp. with exception of *C. krusei*) and absence of deep-seated *Candida* infection received FMGX for a maximum of 14 days (66). Treatment success and survival rates were 80% and 85%, respectively. Another study enrolled 9 patients with *C. auris* candidemia and limited therapeutic options who received FMGX for ≤42 days (67). Most patients (8/9, 89%) responded to therapy and survived. The efficacy of FMGX was also assessed in one trial enrolling patients (*n* = 20) with proven or probable invasive mold infections caused by *Aspergillus* spp. or other molds (*Fusarium* spp., *Scedosporium*/*Lomentospora* spp., Mucorales) with limited therapeutic options (68). Treatment success and overall survival rates were 40% and 75%, respectively.

**TABLE 1:** Phase II and III clinical trials of FMGX^*b*^

First author, year (reference)^*a*^	Study phase (design) *N* participants	IFI type	FMGX (with dosing) and comparator	Outcome
Pappas, 2023 (66)	Phase II (open-label, single-arm) 20 participants	Candidemia	FMGX 1,000 mg bid IV (D1), then 600 mg qd IV and optional switch 700 mg qd PO (from D4) for 14 days	Treatment success 80% Survival (D30) 85%
Vazquez, 2023 (67)	Phase II (open-label, single-arm) 9 participants	Candidemia or IC caused by *C. auris*	FMGX 1,000 mg bid IV (D1), then 600 mg qd IV and optional switch 800 mg qd PO (from D4) for ≤42 days	Treatment success 89% Survival (D30) 89%
Hodges, 2025 (68)	Phase II (open-label, single-arm) 20 participants	Invasive mold infections with limited treatment options	FMGX 1,000 mg bid IV (D1), then 600 mg qd IV and optional switch 800 mg qd PO (from D4) for ≤42 days	Treatment success 40% Survival (D42) 75%
NCT05421858	Phase III (double-blind, two-arm) Recruiting	Candidemia, IC	FMGX IV, then PO (dosing not specified) Caspofungin IV, then fluconazole PO	Treatment success Survival (D30)
NCT06925321	Phase III (open-label, two-arm) Recruiting	Invasive mold infections Primary therapy (cohort A) Salvage therapy (cohort B)	FMGX IV, then PO (dosing not specified) Standard of care antifungal therapy	All-cause mortality (D42)

^*a*^ The clinical trial registration number is provided for ongoing studies.

^*b*^ IFI, invasive fungal infections; IC, invasive candidiasis; IV, intravenous; PO, per os.

Regarding safety, no serious adverse events or drug discontinuations were reported in the two candidemia trials (66, 67). In the invasive mold infections trial, 15/21 (71.4%) participants experienced presumed FMGX-related adverse events (mainly gastrointestinal disorders), which were considered severe in two of them (diarrhea/decreased appetite and sudden death of unclear origin) (68). Seven (33.3%) patients discontinued FMGX, and the cause of interruption was considered drug-related for 3 of them.

Two phase III trials comparing FMGX to standard of care antifungal therapy for IC and invasive mold infections with limited treatment options are ongoing (Table 1). An expanded access program is open for patients with serious or life-threatening IFI and no other treatment options. A preliminary report of 81 patients who received FMGX for invasive fusariosis (mainly *F. solani*) via this program has been recently presented (69). The rates of favorable response were 77.3% and 86.5% in patients with and without hematologic malignancies, respectively. The overall 6 week mortality rate among hematologic patients was 31.8%, which is similar to or lower than those reported in recent cohorts of invasive fusariosis treated with conventional antifungal therapy (70, 71). FMGX was used in combination therapy (in addition to voriconazole and amphotericin B) in seven patients during an outbreak of *Fusarium solani* meningitis following contaminated epidural anesthesia in a Mexican clinic (72). Six of them (85.7%) survived, while the survival rate under conventional antifungal therapy alone (voriconazole and amphotericin B) was much lower (3/11, 27.3%). Some case reports of successful FMGX treatment of refractory fusariosis have also been published (73–75). In a case series of 11 patients with mucormycosis (including three mixed infections: *A. fumigatus*, *Fusarium* spp., and *Curvularia* spp.), FMGX used as primary (*n* = 1) or salvage (*n* = 10) therapy was associated with a 64% favorable response rate (76). Data for other molds are limited. Cobo et al. reported the case of an allogeneic hematopoietic stem cell transplant recipient with *L. prolificans* fungemia who experienced a favorable response to FMGX salvage monotherapy following initial failure of combined antifungal therapy (voriconazole, terbinafine, amphotericin B, miltefosine) (77). Camargo et al. reported a case of disseminated *Aspergillus calidoustus* infection (including endocarditis) who experienced a favorable outcome with rapid serum galactomannan decline following the introduction of FMGX as salvage combination therapy followed by prolonged maintenance therapy (78). Raman and Sharma used FMGX as maintenance therapy to treat a case of extensive musculoskeletal coccidioidomycosis (79).

## RESISTANCE TO FMGX

Reduced susceptibility to MGX could be achieved *in vitro* by micro-evolution protocols in *Candida* spp. (80, 81). Mutations in the target protein (Gwt1) were obtained in *C. albicans* (V162A) and *C. glabrata* (V163A), resulting in 16- to 32-fold MGX MIC increases (81). Dai et al. characterized the Gwt1 binding pocket of MGX and identified several potential resistance mutations in this hotspot (19). Artificial overexpression of *GWT1* was shown to induce MGX resistance, but this mechanism has not yet been observed among clinical isolates (24). Importantly, decreased susceptibility to MGX was observed as a result of overexpression of efflux pumps in *C. albicans* (*CDR11* and *SNQ2*, triggered by the W986L mutation in the transcription factor Zcf29), *Candida parapsilosis* (*MDR1*, resulting from a mitochondrial deletion), and *C. auris* (*CDR1*, triggered by the D865N mutation in the transcription factor Tac1b) (80, 82). Gain-of-function mutations in the transcription factors controlling efflux pump expression represent a common mechanism of azole resistance among *Candida* spp. (83), which suggests the possibility of a cross-resistance to both azoles and MGX. Indeed, antifungal susceptibility testing of large collections of *Candida* isolates showed a correlation between MGX non-wild-type MIC and fluconazole resistance (27, 28, 84). Reduced MGX susceptibility was observed in azole-resistant strains harboring mutations in Tac1 (*C. albicans*, *C. parapsilosis*), Pdr1 (*C. glabrata*), or Tac1b (*C. auris*) (85). This resistance mechanism related to efflux pump overexpression resulted in MGX MIC increase of about 4- to 16-fold, which remains relatively modest considering the very high susceptibility of wild-type *Candida* spp., and its possible clinical impact is still unknown. Indeed, clinical breakpoints have not yet been defined for FMGX. *Post hoc* PK/PD analyses of ongoing phase III clinical trials will be needed to assess clinical breakpoints that may guide antifungal therapy.

Mechanisms of MGX resistance in *Aspergillus* spp. and other molds remain to be investigated.

## NOVEL COMPOUNDS IN THE GEPIX CLASS

Analogs of MGX and FMGX have been synthesized to achieve more potent fungal Gwt1 inhibition, including APX2020 (prodrug APX2097), APX2039 (prodrug APX2096), and APX2041 (prodrug APX2104) (35). APX2039 demonstrated the highest *in vitro* activity against *Cryptococcus* spp. with MICs 32-fold lower than MGX against both *C. neoformans* and *C. gattii* (35). Its prodrug (APX2096) was highly effective in reducing fungal burden in the lung and brain in murine and rabbit models of cryptococcal meningitis with an effect that was equivalent to or superior to that of amphotericin B (35, 86). APX2041 displayed comparable *in vitro* activity to MGX against *A. fumigatus,* and its prodrug APX2104 was effective in murine models of IA (against both wild-type and azole-resistant strains) (35, 87). Both APX2041 and APX2020 were highly active against *Coccidioides* spp. (MIC similar to or lower than MGX), and their respective prodrugs were effective in reducing lung and spleen fungal burden in mice (43). APX2097 was even superior to both fluconazole and FMGX in rescuing mice in a survival model of coccidioidomycosis (43).

## CLINICAL PERSPECTIVES FOR FMGX AND ANALOGS

FMGX and novel compounds of the gepix class offer potential advantages over current antifungal drugs, such as broad-spectrum antifungal activity, oral bioavailability, CNS penetration, and acceptable tolerance. Their main limitation consists of their non-fungicidal activity, which is, however, compensated by potent fungistatic activity and prolonged PAE. Their potential to induce drug resistance, particularly cross-resistance with azoles, may be concerning and should be closely monitored. Overall, FMGX is expected to have an important place in the management of IFIs in the future. Its possible niches will be discussed in the next sections and are summarized in Table 2.

**TABLE 2:** Possible scenarios for use of FMGX for the treatment of invasive fungal infections

Invasive fungal infection	Scenario	Place for FMGX^*a*^	Evidence^*b*^
Invasive candidiasis	First-line therapy	If echinocandin resistance^*c*^^,*d*^	4
		If involvement of urinary tract or CNS/eye^*c*^^,*d*^	2
	Second-line therapy: salvage therapy	If failure of echinocandins^*c*^^,*e*^ Consider combination with echinocandins or amphotericin B	4*^j^*
	Second-line therapy: switch to oral medication	Following initial echinocandin therapy^*c*^^,*d*^	4^*j*^
Cryptococcal meningitis	Second-line therapy: salvage therapy	Following failure or toxicity of azoles or amphotericin B^*f*^^,*g*^ Consider combination therapy (with currently recommended antifungals)	2
	Second-line therapy: switch to oral medication	If azole toxicity^*f*^	2^*j*^
Invasive aspergillosis	First-line therapy	If azole resistance and high risk of amphotericin B toxicity^*g*^	3
	Second-line therapy: salvage therapy	Following failure or toxicity of azoles or amphotericin B*^f^*^,^*^g^*^,^*^h^* Consider combination with amphotericin B or azoles	3
	Second-line therapy: switch to oral medication	If azole resistance or toxicity^*f*^	3^*j*^
Invasive mucormycosis	Second-line therapy: salvage therapy	Following failure or toxicity of both azoles and amphotericin B^*f*^^,*g*^ Consider combination with amphotericin B or azoles	3
	Second-line therapy: switch to oral medication	If azole toxicity^*f*^	3^*j*^
Invasive fusariosis	First-line therapy	May be considered as first choice if isolate susceptible^*i*^	4
	Second-line therapy: salvage therapy	In case of failure or toxicity of azoles or amphotericin B^*f*^^,*g*^ Consider combination with amphotericin B or azoles	4
	Second-line therapy: switch to oral medication	May be considered as first choice^*i*^ or if azole toxicity^*f*^	4^*j*^
Invasive scedosporiosis/ lomentosporiosis	First-line therapy	May be considered as first choice for *L. prolificans* If azole toxicity^*f*^ (*S. apiospermum*)	2
	Second-line therapy: salvage therapy	Following failure or toxicity of azoles^*f*^ Consider combination with azoles	2^*j*^
	Second-line therapy: switch to oral medication	May be considered as first choice (*L. prolificans*) or if azole toxicity^*f*^ (*S. apiospermum*)	2^*j*^
Coccidioidomycosis	Second-line therapy: salvage therapy	Following failure or toxicity of azoles^*f*^	2
	Second-line therapy: switch to oral medication	If azole toxicity^*f*^	2^*j*^
Other (rare) yeast or mold infections	Second-line therapy: salvage therapy	If FMGX *in vitro* activity demonstrated and no other alternatives (because of resistance or toxicity of conventional antifungals)	1
	Second-line therapy: switch to oral medication	If FMGX *in vitro* activity demonstrated and no other alternatives (because of resistance or toxicity of conventional antifungals)	1

^*a*^ Perspectives and possible applications for the future use of FMGX in clinical practice on the basis of the author’s appreciation. These suggestions cannot be considered as recommendations unless evidence from phase III clinical trials has supported these statements.

^*b*^ Evidence is rated as follows: 1. *In vitro* data only, 2. Data derived essentially from animal models (no clinical data or limited to case reports, *n* < 5) 3. Small case-series (5 to 20 cases), 4. Data from small phase II clinical studies or case-series (*n* ≥ 20 cases). 5. Data from large comparative phase II or III clinical studies. For references supporting these levels of evidence, refer to text.

^*c*^ Except *C. krusei*, *C. kefyr*, *C. inconspicua*.

^*d*^ Pending results of randomized trials (and demonstration of FMGX non-inferiority), azoles (mainly fluconazole) should be preferred if the isolate is susceptible and in the absence of toxicity (or risk of toxicity).

^*e*^ Failure to echinocandins is a rare scenario. Consider other causes of failure (e.g., insufficient source control).

^*f*^ Azole toxicity (or risk of toxicity): moderate/severe hepatic insufficiency, QT prolongation (except for isavuconazole).

^*g*^ Amphotericin B toxicity (or risk of toxicity): renal insufficiency, severe electrolyte imbalances, severe infusion-related reactions.

^*h*^ Pending results of randomized trials (and demonstration of FMGX non-inferiority), amphotericin B lipid formulation (in case of initial azole therapy) or azoles (in case of initial amphotericin B therapy) should be preferred as salvage therapy.

^*i*^ In particular for *F. solani* isolates exhibiting high azoles and amphotericin B MICs and low MGX MIC.

^*j*^ Specific data for this scenario (i.e. second-line therapy) are lacking. The level of evidence is inferred from data of first-line therapy.

### IC

Echinocandins are expected to remain the first-choice therapy for IC because of their excellent tolerance and broad-spectrum anti-*Candida* fungicidal activity. FMGX may provide some advantages over echinocandins that are similar to fluconazole, such as large tissue distribution and oral formulations for prolonged therapy. Of note, despite good penetration of MGX in CNS/eye, urine, and abdominal abscesses in animal models (46, 53, 54), data on clinical efficacy in humans for these deep-seated infections are lacking.

Because echinocandin and azole resistance is an increasing problem among *Candida* spp., FMGX may represent an alternative option for both first-line or step-down therapy for resistant IC. In this scenario, it will enter into competition with ibrexafungerp, which offers similar advantages (i.e., broad-spectrum anti-*Candida* activity, including against echinocandin- or azole-resistant isolates, and oral bioavailability) (16, 88). Data on FMGX efficacy for IC are currently limited to small phase II studies, which were restricted to highly selected patients (e.g., absence of neutropenia, exclusion of deep-seated infections) (66, 67). The position of FMGX for the treatment of IC will be better defined after completion of the phase III trial comparing its efficacy with standard of care antifungal therapy for IC (Table 1). Its poor activity against some *Candida* spp. (*C. krusei*, *C. kefyr*, *C. inconspicua*) should be kept in mind for use as empirical antifungal therapy for IC, although these species are relatively uncommon. Moreover, the clinical relevance of cross-resistance of FMGX with azoles among *Candida* spp. should be assessed in the future and could represent a major limitation for its use in this setting.

### Cryptococcosis

FMGX may have a place in the treatment of cryptococcosis, in particular cryptococcal meningitis. It could be considered as salvage therapy in combination with currently recommended first-line therapies or as an alternative to fluconazole for maintenance therapy. The novel compound APX2096 (prodrug of APX2039) may display more potential than FMGX for future application in the treatment of cryptococcosis based on pre-clinical studies (35, 86). Clinical studies are needed to demonstrate its non-inferiority compared to amphotericin B ± flucytosine or fluconazole. In this setting, it may be in competition with oral lipid nanocrystal amphotericin B, which demonstrated comparable efficacy to intravenous amphotericin B in a phase II open-label study (89).

### IA

Data on FMGX for the treatment of IA are currently scarce, being limited to a small phase II study including mixed invasive mold infections with lack of characterization of the fungal pathogens in most of them (68). FMGX may represent an alternative option for the treatment of azole-resistant or refractory IA or in patients with drug-related toxicity to azoles or amphotericin B. Considering the fungistatic effect of FMGX, its non-inferiority compared to the current fungicidal standard of care (azoles and amphotericin B formulations) remains to be demonstrated in clinical trials. Its efficacy and safety will also need to be compared to olorofim, another novel antifungal drug with similar advantages, including activity against azole-resistant *Aspergillus* spp., oral bioavailability, and acceptable CNS penetration (16, 88). Olorofim has already been tested in a single-arm phase II study and showed a response rate (including stable disease) of 65% and a survival rate of 81% at day 42 for refractory IA (*n* = 101) (90). Such large data sets are currently lacking for FMGX.

### Other invasive mold infections

Despite limited *in vitro* activity against *Mucorales*, FMGX demonstrated interesting results in mucormycosis murine models (58, 60). However, clinical data are scarce (68). Preliminary results of a small case series of patients who mainly received FMGX as salvage therapy should be interpreted cautiously. The discrepancy between *in vitro* activity and *in vivo* efficacy in mucormycosis may be due to the multiplicity of parameters affecting outcomes (fungal burden, surgery, neutrophil recovery). Of note, similar observations have been made for posaconazole and isavuconazole (91).

Other rare mold infections with limited therapeutic options, such as invasive fusariosis and scedosporiosis/lomentosporiosis, may represent the main field of application for FMGX based on encouraging preliminary results, particularly for fusariosis (68, 69). Because FMGX has been mainly administered via an expanded access program, which may include a substantial proportion of second-line and/or combination therapies, further trials assessing its efficacy as primary monotherapy will be required. Moreover, the efficacy of FMGX in this setting will need to be confronted with that of olorofim. While olorofim displays variable *in vitro* activity against *Fusarium* spp., it is very active against *Scedosporium*/*Lomentospora* spp. (92–94). Its success rate (including stable disease) for invasive scedosporiosis/lomentosporiosis was 81% at day 42 (*n* = 48), while clinical data for FMGX are very limited in this setting (68, 90).

### Other mycoses

The efficacy of FMGX for the treatment of infections due to rare yeasts that are intrinsically resistant to echinocandins and fluconazole (e.g., *Magnusiomyces* spp., *Trichosporon* spp., *Rhodotorula* spp.) deserves further investigation considering its good *in vitro* activity against many of these species, except *Trichosporon* spp. (29). FMGX may also have a role in the treatment of endemic mycoses, particularly coccidioidomycosis, but experience is still very limited (79). Finally, there are currently no clinical data for localized mycoses, in particular vulvovaginal or oral candidiasis. Considering that multi-resistant *Candida* spp. are often encountered in cases of recurrent infections, FMGX could represent an interesting alternative to azoles for oral therapy.

## CONCLUSIONS

The first-in-class gepix compound FMGX is a promising novel antifungal drug with broad-spectrum antifungal activity against most pathogenic fungi, including notoriously resistant species (e.g., *C. auris*, azole-resistant *A. fumigatus*, *Fusarium* spp., and *Scedosporium*/*Lomentospora* spp.) and improved pharmacologic properties (oral bioavailability, CNS penetration, low toxicity). Current clinical data are limited by the small size of data sets, the lack of comparative trials, the heterogeneity of IFI types and host underlying conditions, as well as the potential bias of data derived from patients having received FMGX as salvage and/or combination therapy via the expanded access program. The place of FMGX in the therapeutic strategies of IFIs will need to be better defined based on the results of ongoing phase III trials. Because FMGX is a fungistatic drug, demonstration of its non-inferiority compared to echinocandins for the treatment of IC and compared to mold-active azoles and amphotericin B for IA are important steps to achieve. Difficult-to-treat mold infections, such as azole-resistant IA, fusariosis, and scedosporiosis/lomentosporiosis, definitely represent the setting where FMGX may become a privileged salvage therapy or even a first-line therapeutic option. Importantly, the respective places of FMGX and olorofim for mold infections, and FMGX and ibrexafungerp for *Candida* infections will need to be better defined. Finally, novel compounds in the gepix class may find their niches in the future, such as APX2039 (prodrug APX2096) for cryptococcal meningitis and APX2020 (prodrug APX2097) for coccidioidomycosis. Resistance to FMGX, in particular cross-resistance with azoles via overexpression of efflux pumps, is expected to arise. Epidemiological surveys of resistance and assessment of susceptibility-outcome relationships will be needed to better define the place of FMGX in the future.
